# Sepsis in burn care: incidence and outcomes

**DOI:** 10.1186/s40779-025-00643-x

**Published:** 2025-09-01

**Authors:** Diana Julia Tedesco, Maria Fernanda Hutter, Fadi Khalaf, Zachary Ricciuti, Marc G. Jeschke

**Affiliations:** 1 Department of Biochemistry and Biomedical Sciences, Faculty of Health Sciences, McMaster University, Hamilton, ON L8S 4K1 Canada; 2 Centre for Burn Research, Hamilton Health Sciences, Hamilton, ON L8L 2X2 Canada; 3 David Braley Research Institute, Hamilton, ON L8L 2X2 Canada; 4 Division of Plastic and Reconstructive Surgery, Department of Surgery, McMaster University, Hamilton, ON L8S 4K1 Canada; 5 Division of Plastic and Reconstructive Surgery, Department of Surgery, Medical University of Graz, 8036 Graz, Austria; 6 Department of Medical Sciences, Faculty of Health Sciences, McMaster University, Hamilton, ON L8S 4K1 Canada

**Keywords:** Burn, Sepsis, Mortality, Infection, Female, Trauma

## Abstract

**Background:**

Although sepsis is known to be the leading cause of morbidity and mortality in adult burn patients, its epidemiology and impact are poorly understood. This study aims to address these gaps by further characterizing predictors of sepsis and comparing outcomes between septic and non-septic burn patients in different age groups.

**Methods:**

We included patients (≥ 18 years) with thermal burn injuries ≥ 5% total body surface area (TBSA) admitted to two burn centers between 1 January 2006 and 30 June 2021, and 1 January 2023 and 6 April 2025. Patients were stratified by age into adults (18–59 years) and older adults (≥ 60 years), and by diagnosis of sepsis during hospitalization (sepsis vs. control). Demographics, injury characteristics, mortality, and in-hospital complications were assessed. Multivariate logistic regression models were used to identify predictors of sepsis and mortality among septic patients.

**Results:**

This study included a total of 1465 patients, including 1094 adults and 371 older adults. Sepsis was diagnosed in 20.1% of adult burn patients, with a median onset at 10 d following injury. Increasing age, greater TBSA, and inhalation injury were identified as significant risk factors for sepsis. Among patients who developed sepsis, earlier onset and female sex were associated with an elevated risk of mortality. In older adults, the incidence of sepsis was 22.9%, with a median onset at 11 d post-burn. The odds of sepsis diagnosis increased with higher TBSA and the presence of inhalation injury. Earlier sepsis onset was associated with increased mortality in older adults.

**Conclusions:**

Sepsis represents a significant clinical challenge in burn patients, with age, TBSA, inhalation injury, and comorbidities significantly influencing its incidence and outcomes. Notably, early sepsis onset and female sex are associated with increased mortality, highlighting the need for advanced monitoring, prompt interventions, and the exploration of innovative sex-specific strategies to optimize outcomes in this high-risk population.

**Supplementary Information:**

The online version contains supplementary material available at 10.1186/s40779-025-00643-x.

## Background

Burn injuries impact 9 million people worldwide each year [1] and account for up to 20% of casualties in post-World War II conflicts [2]. Among military personnel, 18% of burn patients develop infections, with 69% experiencing multiple infections [3]. As burn injuries severely compromise the skin, the body’s primary defense against microbial invasion, the risk of infection increases greatly, especially in those with ≥ 20% total body surface area (TBSA) burns, where the rate of infection has been reported to be as high as 55% [4]. Additionally, the limited resources and delays of care in combat zones elevate the risk of infectious complications [5], which in turn contributes to the high prevalence of sepsis among military burn patients. Indeed, the incidence of sepsis in patients with burns covering ≥ 20% of their TBSA ranges from 3 to 30%, with some studies reporting rates as high as 39% [6–8]. Despite significant advancements in the management and treatment of burn injuries, sepsis remains the leading cause of death among burn patients [9]. Although sepsis is a significant concern, its actual incidence, clinical characteristics, and outcomes in burn patients are not well-documented due to studies with small populations and limited data.

As defined by the Sepsis-3 guidelines, sepsis is a life-threatening organ dysfunction which occurs due to an abnormal and dysregulated host response to infection [10]. This response leads to a range of adverse effects, including hemodynamic instability, inhibition of mitochondrial respiration, and increased capillary permeability resulting in tissue edema and hypovolemia, and a shift toward a procoagulant state [11–14]. In some cases, these circulatory, cellular, and metabolic abnormalities will worsen beyond the typical septic response, leading to septic shock, a state that is associated with a higher risk of mortality than sepsis alone [10]. Importantly, according to the 2007 American Burn Association (ABA) guidelines for diagnosing sepsis in burn patients, these alterations overlap with those seen in the acute post-burn hypermetabolic response, resulting in similar physical symptoms, including tachycardia (> 110 beats/min), progressive tachypnea (> 25 breaths/min), and thrombocytopenia (platelet count < 100 × 10^3^/µl) as key diagnostic criteria [9, 15, 16]. Therefore, the similarities between traditional signs of sepsis and post-burn hypermetabolism have consistently posed challenges in the early diagnosis of burn-induced sepsis. Although the adoption of early excision has significantly reduced the incidence of wound infections, the inability to achieve timely diagnosis and treatment of sepsis undermines these advancements, limiting the progress made in burn care over the past decades [12].

Recent efforts have focused on refining the definition and diagnostic criteria of sepsis to incorporate advancing knowledge and improve its detection in burn patients. However, meaningful improvements in its early identification are yet to be seen. A comparison of the scoring systems currently used for sepsis diagnosis in burn patients revealed that they lack the sensitivity and specificity necessary to accurately and reliably predict sepsis in burn patients [17]. Hence, the efforts to enhance early diagnosis will not yield improvements in patient outcomes unless we deepen our understanding of the characteristics of burn-induced sepsis.

Moreover, despite the stark differences in outcomes between adult and older adult burn patients, there is limited research that describes the epidemiological distinctions between these two groups. Indeed, older adults have been shown to exhibit altered inflammatory and immune responses following burn injuries, which can significantly impact infection risk and outcomes [18]. As a result, older adult burn patients have a significant increase in mortality and multi-organ failure, delayed initiation of the hypermetabolic response, immune suppression, and substantial delays in wound healing [18, 19]. Despite the heightened vulnerability of this population, specific data pertaining to sepsis diagnosis and outcomes in older adult burn patients are scarce, indicating that improvements in its diagnosis are needed to help mitigate the already elevated risks of mortality.

This study aims to characterize sepsis incidence and outcomes within a large cohort of adult and older adult burn patients to highlight the critical demographic, clinical, and temporal factors that influence its onset and outcomes. By thoroughly describing this population, we aim to identify key patterns that could inform more effective, timely identification, and improve outcomes for septic burn patients, ultimately reducing mortality and complications associated with delayed diagnosis.

## Methods

This study adhered to the Strengthening the Reporting of Observational Studies in Epidemiology statement [20].

### Study participants

This retrospective cohort study involved adult patients admitted to the Ross Tilley Burn Centre at Sunnybrook Health Sciences Centre in Toronto, Canada, between 1 January 2006 and 30 June 2021, and the Hamilton Health Sciences Burn Unit in Hamilton, Canada, between 1 January 2023 and 6 April 2025. All patients received standard-of-care treatment for their injuries according to standardized clinical protocols, which included early excision and grafting, early nutrition, adequate antibiotic coverage, and ventilation, as needed. This study received approvals from the Sunnybrook Research Ethics Board (307-2015) and Hamilton Integrated Research Ethics Board (16705), and informed consent were waived.

Eligible patients were selected for inclusion based on meeting the following criteria: adult patients (≥ 18 years of age) admitted for an acute burn injury with a recorded TBSA ≥ 5%. Patients with non-burn injuries, with burns of non-thermal etiology (electrical, chemical, or other), admitted for non-acute injuries, missing recorded TBSA burn, and who died within 3 d post-burn were excluded. Patients who died within 72 h post-burn were considered “futile cases”, as early mortality may reflect overwhelming disease severity that precludes meaningful intervention, to prevent biased assessments of prognostic associations. As age-dependent differences are known to affect post-burn outcomes, we then stratified included patients based on age: adults (18–59 years) and older adults (≥ 60 years). We defined “older adults” as individuals aged 60 years and above, in accordance with the World Health Organization classification, which recognizes this age group as being at an increased risk for age-related complications and adverse outcomes [21].

### Data sources and variables

Demographics and clinical outcomes were recorded prospectively by the burn team, including the attending burn and critical care staff, through daily rounds. Outcomes included the number of septic episodes, days to the onset of the first sepsis episode, operative procedures, mechanical ventilation, and 30-day in-hospital mortality.

To assess sepsis outcomes within each age group, patients who experienced at least one episode of sepsis during hospitalization were compared to non-sepsis controls. Sepsis was defined prospectively by the attending burn and critical care staff and multidisciplinary burn team based on a combination of clinical symptoms and laboratory findings, in accordance with established guidelines. Specifically, sepsis identification followed the ABA sepsis guidelines [16], which were tailored to the burn population and account for the unique physiological responses to burn injury. In addition, the Sepsis-3 criteria [10] were applied, incorporating the presence of suspected or confirmed infection and an acute increase in the sequential organ failure assessment score by ≥ 2 points from baseline.

Several in-hospital complications were also assessed prospectively by the burn care team. Acute kidney injury (AKI) was diagnosed using the Kidney Disease: Improving Global Outcomes (KDIGO) guideline [22]. Acute respiratory distress syndrome (ARDS) was identified using the Berlin definition, requiring bilateral infiltrates on imaging, hypoxemia, and absence of cardiogenic causes [23]. Differentiation from pneumonia was primarily based on radiologic pattern (diffuse vs. focal), the absence of purulent secretions, and the lack of microbiologic evidence of infection. Close monitoring and daily rounds enabled a comprehensive assessment of the patient and a reliable diagnosis of any complications, ensuring timely and adequate medical interventions. The length of stay was calculated for survivors and defined as the number of days elapsed between admission and discharge from the Burn Centre. Additionally, the number of days to the onset of the first sepsis diagnosis was compared. Pathogens recorded for the first sepsis diagnosis were compared by their genus and Gram stain classification: Gram-positive, Gram-negative, or both (Gram-positive and Gram-negative).

### Statistical analysis

Descriptive statistics were used to summarize patient demographics, injury characteristics, pre-admission comorbidities, 30-day mortality, and in-hospital complications. Continuous variables were presented as mean ± standard deviation for normally distributed variables or median [interquartile range (IQR)] for non-normally distributed variables, and compared using unpaired Student’s *t*-test or Mann-Whitney *U* test, as appropriate. Normality was assessed using the Kolmogorov-Smirnov test. Categorical variables were presented as *n* (%) and compared using Fisher’s exact test. For multiple comparisons, the Kruskal-Wallis test was used to compare continuous variables and the Dunn’s correction was applied to adjust the significance threshold. Multiple comparisons with categorical variables were conducted using the *χ*^2^ test.

Kaplan-Meier survival analyses were conducted to compare time-to-mortality up to 30 d post-admission between groups. Differences in survival distributions were tested using the log-rank (Mantel-Cox) test and reported as the hazard ratio (*HR*) and 95% confidence interval (CI).

Univariate logistic regression was initially performed to assess the association between the first episode of sepsis and individual clinical and demographic variables, including age, sex, TBSA, third-degree TBSA, inhalation injury, and preadmission comorbidities. To account for the combined effect of these risk factors, multivariate logistic regression was conducted to examine the association between sepsis diagnosis and 5 variables selected based on clinical relevance and prior literature: age, sex, TBSA, third-degree TBSA, and inhalation injury.

Additionally, univariate logistic regression was performed to examine predictors of mortality among patients who developed sepsis, which included days to the onset of the first episode of sepsis, age, sex, TBSA, third-degree TBSA, inhalation injury, and preadmission comorbidities as candidate covariates. Days to the first sepsis episode, age, sex, TBSA, third-degree TBSA, and inhalation injury were entered into the multivariate logistic regression model.

As our primary objective was to estimate the main effects of clinically relevant predictors on sepsis diagnosis and mortality, we did not include interaction terms in the multivariate models. Model performance was evaluated using the Omnibus Tests of Model Coefficients to determine the overall significance of each model. Additionally, model fit was assessed using the Hosmer and Lemeshow goodness-of-fit test, and the Nagelkerke *R*^2^ was reported as an estimate of the proportion of variance explained by the model. Unadjusted and adjusted odds ratios (*OR*s) with 95% CI were reported for independent variables.

Statistical comparisons were performed using IBM SPSS Statistics Version 30.0.0 (Armonk, NY, USA). Survival analyses and figure generation were conducted using GraphPad Prism version 10 (Boston, MA, USA). Statistical tests were two-tailed, with *P* < 0.05 considered statistically significant.

## Results

During the study period, 3258 admitted patients were assessed for eligibility, and 1793 patients were excluded due to age (*n* = 39), non-burn injuries (*n* = 269), non-thermal etiology (*n* = 250), non-acute cases (*n* = 138), missing TBSA (*n* = 174), TBSA < 5% (*n* = 826), and death within 72 h of admission (*n* = 97). A total of 1465 patients were confirmed eligible and included in the final analysis, including 220 adult sepsis, 874 adult control, 85 older adult sepsis, and 286 older adult control patients (Additional file 1: Fig. S1).

### Sepsis diagnosis and outcomes in adult burn patients

**Fig. 1:**
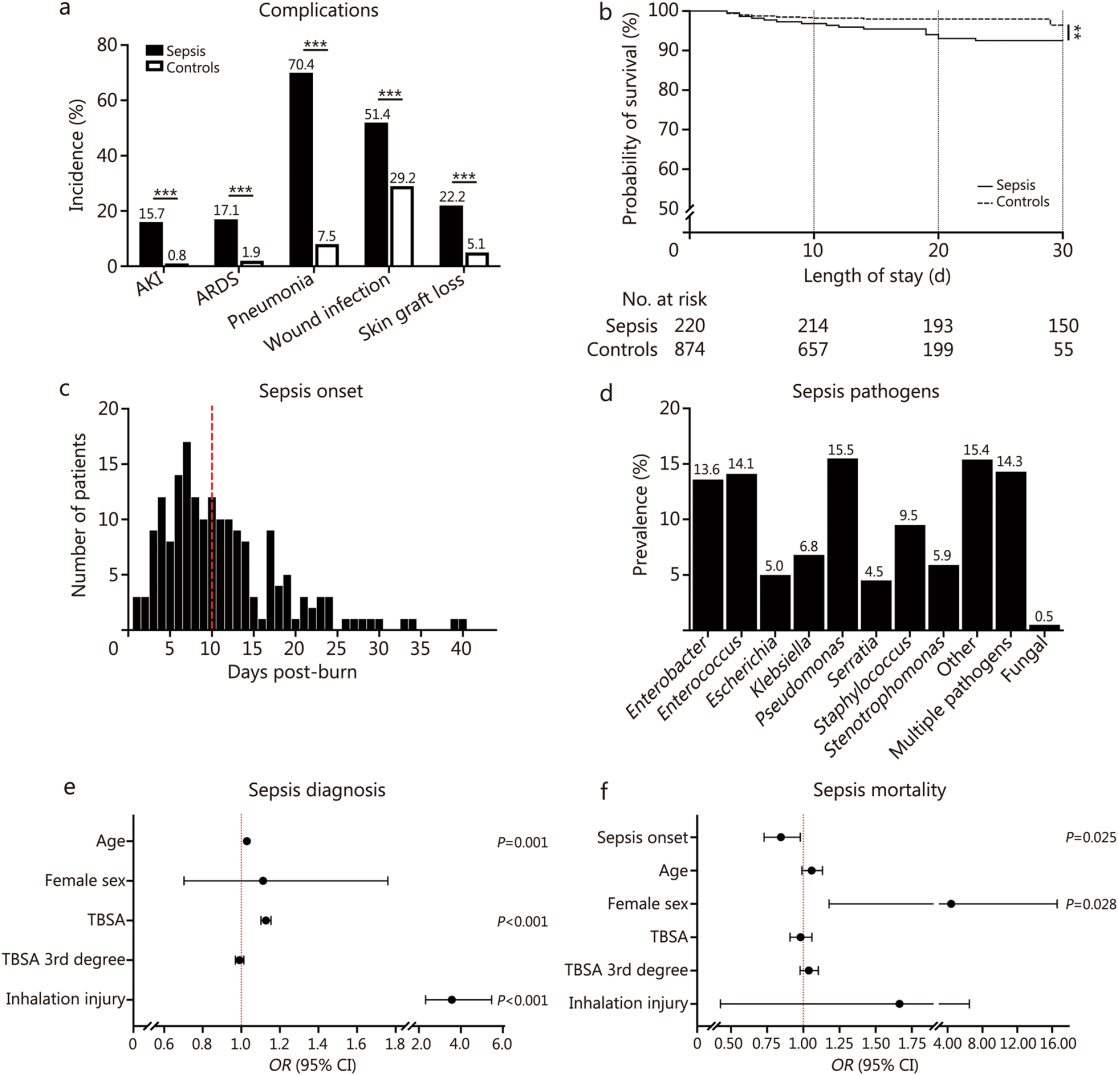
Sepsis-related characteristics, complications, and outcomes in adult burn patients. **a** Overview of complication rates among septic and non-septic patients during hospitalization. **b** Kaplan–Meier survival analysis comparing 30-day post-burn survival between septic and non-septic patients. **c** Distribution of the timing of the first sepsis diagnosis following burn injury; red dashed line indicates the median onset at 10 d post-burn. **d** Microbial profile of bacterial and fungal pathogens isolated at the time of sepsis diagnosis. **e** Multivariate analysis identifying patient and injury factors associated with an increased odds of sepsis diagnosis. **f** Multivariate model examining predictors of mortality in septic patients. ^**^P < 0.01, ^***^P < 0.001. AKI acute kidney injury, ARDS acute respiratory distress syndrome, CI confidence interval, TBSA total body surface area, *OR* odds ratio

Among adults, 20.1% of burn patients were diagnosed with sepsis during their hospitalization. Demographics, injury characteristics, pre-admission comorbidities, and outcomes of adult burn patients were presented in Table 1. Sepsis patients experienced increased 30-day mortality (7.3% vs. 1.8%, *P* < 0.001) and in-hospital complications compared with controls, including increased AKI (15.7% vs. 0.8%, *P* < 0.001), ARDS (17.1% vs. 1.9%, *P* < 0.001), pneumonia (70.4% vs. 7.5%, *P* < 0.001), wound infection (51.4% vs. 29.2%, *P* < 0.001), and skin graft loss (22.2% vs. 5.1%, *P* < 0.001) (Table 1; Fig. 1a). Survival was lower among sepsis patients compared to controls with a *HR* of 2.39 (95% CI 1.12–5.12, *P* = 0.008; Fig. 1b). Among adult patients, the first episode of sepsis was clinically diagnosed at a median (IQR) of 10 (6–14) d post-burn (Fig. 1c).

**Table 1:** Demographics, injury characteristics, and outcomes of adult sepsis and control burn patients

Characteristics	Sepsis (*n* = 220)	Controls (*n* = 874)	*P-*value
Demographics			
Age [years, median (IQR)]	44.0 (35.0–52.0)	40.0 (28.0–49.0)	< 0.001
Sex [*n* (%)]			0.799
Male	163 (74.1)	639 (73.1)	
Female	57 (25.9)	235 (26.9)	
Injury characteristics			
Burn etiology [*n* (%)]			
Flame	188 (85.5)	530 (60.6)	< 0.001
Scald	17 (7.7)	250 (28.6)	< 0.001
Other	15 (6.8)	94 (10.8)	0.101
TBSA [%, median (IQR)]	32.0 (21.0–47.0)	10.0 (7.0–15.0)	< 0.001
TBSA 3rd degree [%, median (IQR)]	16.8 (2.0–36.1)	0.5 (0.0–5.0)	< 0.001
Inhalation injury [*n* (%)]	117 (53.2)	109 (12.5)	< 0.001
Pre-admission comorbidities^a^			
Hypertension [*n* (%)]	31 (14.4)	88 (10.5)	0.118
Diabetes mellitus [*n* (%)]	16 (7.4)	49 (5.9)	0.428
Respiratory disease [*n* (%)]	14 (6.5)	46 (5.5)	0.621
Current smoker [*n* (%)]	59 (27.3)	248 (29.7)	0.557
Alcoholism [*n* (%)]	54 (25.0)	133 (15.9)	0.003
Illicit drug use [*n* (%)]	57 (26.4)	129 (15.4)	< 0.001
Major psychiatric illness [*n* (%)]	60 (27.8)	120 (14.4)	< 0.001
Outcomes			
Operation count [median (IQR)]^a^	4.0 (2.0–7.0)	1.0 (0.0 – 2.0)	< 0.001
Operation and procedure count [median (IQR)]^a^	13.0 (6.0–24.0)	3.0 (0.0 – 5.0)	< 0.001
Ventilation [*n* (%)]^a^	192 (88.9)	195 (23.3)	< 0.001
30-day mortality [*n* (%)]	16 (7.3)	16 (1.8)	< 0.001
LOS [d, median (IQR)]^b^	44.0 (28.0–68.0)	14.0 (10.0–19.0)	< 0.001

^a^Data presented for 1052 patients (216 sepsis and 836 controls)

^b^Analysis restricted to patients alive until discharge. Numbers may not add to 100 due to rounding

*IQR* interquartile range, *LOS* length of stay, *TBSA* total body surface area

*Pseudomonas* (15.5%), *Enterococcus* (14.1%), and *Enterobacter* (13.6%) were most commonly identified at the time of sepsis diagnosis. Approximately 14.3% of adult patients presented with multiple pathogens at the time of sepsis diagnosis, with 0.5% of patients experiencing fungal sepsis (Fig. 1d). Indeed, a comparison of infectious pathogens based on Gram stain showed that most patients presented with Gram-negative sepsis (64.1%). Median (IQR) of TBSA was highest among patients with both Gram-negative and Gram-positive sepsis [48.5 (35.5–57.0) %] compared to only Gram-negative [34.3 (25.3–47.4) %, *P* = 0.034] or Gram-positive [30.0 (20.0–45.8) %, *P* = 0.012] (Additional file 1: Table S1). However, infectious pathogens did not influence survival (Additional file 1: Fig. S2).

### Predictors of sepsis onset in adult burn patients

Univariate logistic regression analyses were conducted to identify independent predictors of sepsis (Additional file 1: Table S2). Age was a significant predictor, with each additional year associated with a 3% increase in the odds of sepsis (*OR* = 1.03, 95% CI 1.01–1.04, *P* < 0.001). TBSA was one of the strongest predictors, with each 1% increase in TBSA associated with a 14% increase in sepsis risk (*OR* = 1.14, 95% CI 1.12–1.15, *P* < 0.001). Similarly, each 1% increase in third-degree TBSA was significantly associated with the increase in sepsis risk (*OR* = 1.09, 95% CI 1.08–1.11, *P* < 0.001). Inhalation injury was also strongly predictive, with patients having nearly 8-times the odds of sepsis during hospitalization (*OR* = 7.97, 95% CI 5.72–11.11, *P* < 0.001).

Among comorbidities, alcoholism (*OR* = 1.76, 95% CI 1.23–2.52, *P* = 0.002), illicit drug use (*OR* = 1.97, 95% CI 1.38–2.81, *P* < 0.001), and major psychiatric illness (*OR* = 2.30, 95% CI 1.61–3.27, *P* < 0.001) were all associated with increased sepsis risk, although their contributions to explained variance were smaller (Nagelkerke *R*^2^ = 0.013–0.029). In contrast, female sex, hypertension, diabetes, respiratory disease, and smoking status were not predictors of sepsis in univariate models.

Multiple logistic regression identified increasing age, greater TBSA, and the presence of inhalation injury as significant predictors of sepsis, whereas female sex and third-degree TBSA showed no association (Fig. 1e). Indeed, each one-year increase in age was associated with a 3% increase in the odds of developing sepsis (*OR* = 1.03, 95% CI 1.01–1.05, *P* = 0.001). Likewise, each 1% increase in TBSA corresponded with a 13% increase in the odds of sepsis (*OR* = 1.13, 95% CI 1.10–1.15, *P* < 0.001). Finally, the presence of inhalation injury significantly increased the odds of sepsis by more than 3-fold (*OR* = 3.57, 95% CI 2.32–5.50, *P* < 0.001). Overall, the model demonstrated good performance, distinguishing between patients with and without sepsis (Omnibus *χ*^2^ = 446.628, *P* < 0.001), explaining 53% of the variance in sepsis diagnosis (Nagelkerke *R*^2^ = 0.533). Model calibration indicated borderline fit (Hosmer-Lemeshow *χ*^2^ = 15.581, *P* = 0.049).

### Predictors of mortality in adult burn patients diagnosed with sepsis

Univariate logistic regression analyses were conducted to examine the associations between various independent variables and mortality among adult patients diagnosed with sepsis (Additional file 1: Table S3). Each additional day to the first sepsis episode decreased the odds of mortality by 17% (*OR* = 0.83, 95% CI 0.72–0.96, *P* = 0.013). Female sex increased the odds of mortality by 4-fold (*OR* = 4.18, 95% CI 1.48–11.81, *P* = 0.007). Additionally, each 1% increase in third-degree TBSA significantly increased the odds of mortality by 3% (*OR* = 1.03, 95% CI 1.01–1.06, *P* = 0.008).

In the multivariate model, the earlier onset of the first sepsis episode and female sex were associated with mortality risk, whereas age, TBSA, third-degree TBSA, and inhalation injury showed no association (Fig. 1f). Each additional day in sepsis onset was associated with a 15% decrease in the odds of mortality (*OR* = 0.85, 95% CI 0.73–0.98, *P* = 0.025), while female patients had a 4-fold increase in the odds of mortality compared to males (*OR* = 4.42, 95% CI 1.18–16.58, *P* = 0.028). Overall, the model demonstrated good performance, distinguishing between survivors and non-survivors (Omnibus *χ*^2^ = 22.564, *P* < 0.001), explaining 29% of the variance in mortality (Nagelkerke *R*^2^ = 0.292), and showing good fit (Hosmer-Lemeshow *χ*^2^ = 7.446, *P* = 0.489).

### Sepsis diagnosis in older adult burn patients

**Fig. 2:**
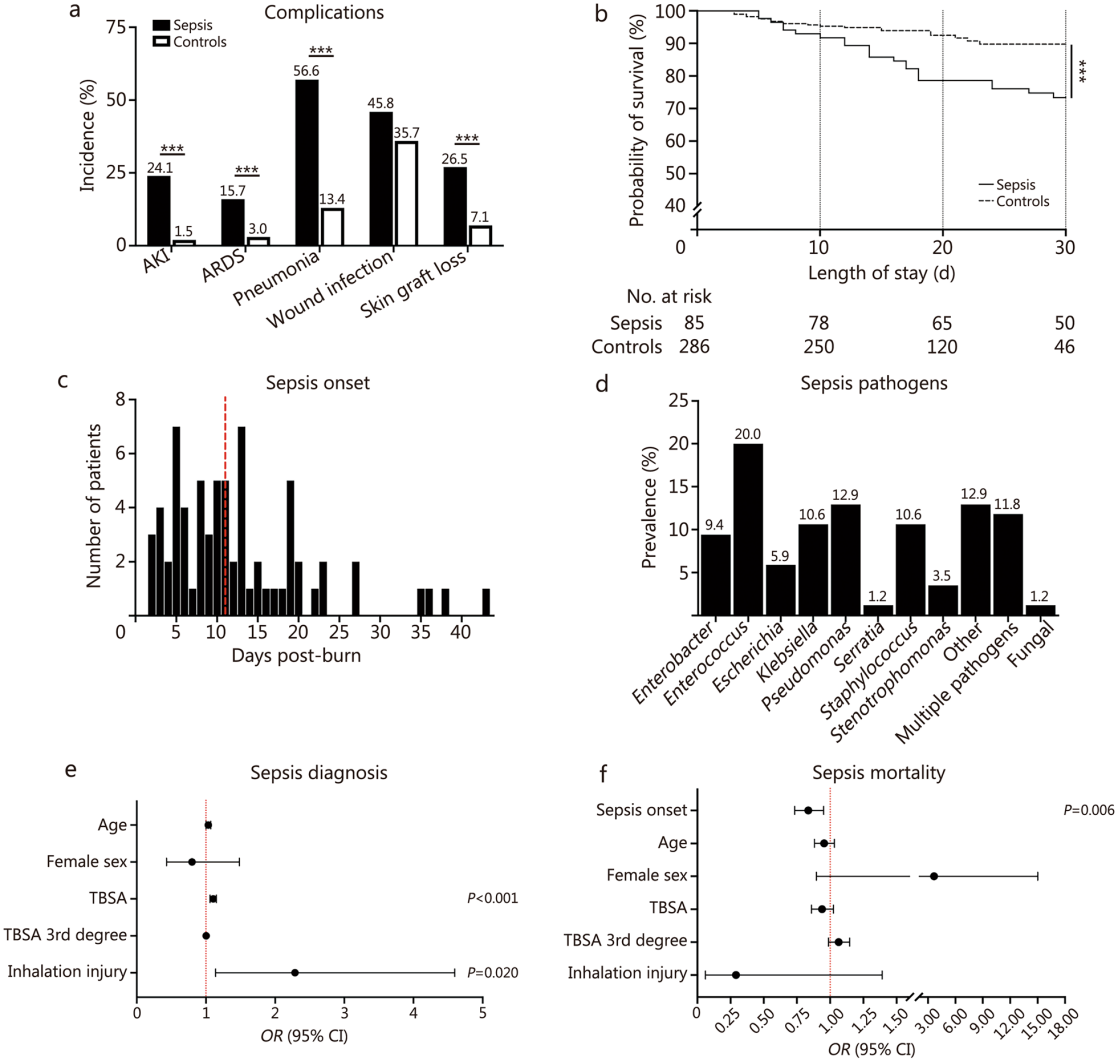
Sepsis-related characteristics, complications, and outcomes in older adult burn patients. **a** Comparison of the incidence of in-hospital complications between septic and non-septic patients. **b** Kaplan–Meier survival analysis comparing 30-day post-burn survival among septic and non-septic patients. **c** Timing of the first sepsis diagnosis post-burn; red dashed line indicates a median onset of 11 d post-burn. **d** Distribution of bacterial and fungal pathogens isolated at the time of sepsis diagnosis. **e** Multivariate analysis assessing predictors of sepsis diagnosis. **f** Multivariate model evaluating predictors of mortality in sepsis patients. ^***^P < 0.001. AKI acute kidney injury, ARDS acute respiratory distress syndrome, CI confidence interval, TBSA total body surface area, *OR* odds ratio

In this cohort, 22.9% of older adult burn patients were diagnosed with sepsis during hospitalization. Demographics, injury characteristics, pre-admission comorbidities, and outcomes of older adult burn patients were presented in Table 2. Sepsis patients experienced increased 30-day mortality (25.9% vs. 7.3%, *P* < 0.001) and incidence of AKI (24.1% vs. 1.5%, *P* < 0.001), ARDS (15.7% vs. 3.0%, *P* < 0.001), pneumonia (56.6% vs. 13.4%, *P* < 0.001), and skin graft loss (26.5% vs. 7.1%, *P* < 0.001) compared with controls (Table 2; Fig. 2a). However, incidences of wound infection (45.8% vs. 35.7%, *P* = 0.120) were similar between sepsis and controls. The probability of survival was lower among sepsis patients compared to controls, with an *HR* of 2.60 (95% CI 1.34–5.04, *P* < 0.001; Fig. 2b).

**Table 2:** Demographics, injury characteristics, and outcomes of older adult sepsis and control burn patients

Characteristics	Sepsis (*n* = 85)	Controls (*n* = 286)	*P-*value
Demographics			
Age [years, median (IQR)]	71.0 (63.0–80.0)	71.0 (64.0–78.0)	0.832
Sex [*n* (%)]			0.512
Male	60 (70.6)	190 (66.4)	
Female	25 (29.4)	96 (33.6)	
Injury characteristics			
Burn etiology [*n* (%)]			
Flame	67 (78.8)	184 (64.3)	0.012
Scald	14 (16.5)	77 (26.9)	0.061
Other	4 (4.7)	25 (8.7)	0.259
TBSA [%, median (IQR)]	22.0 (14.0–31.0)	10.4 (7.0–16.1)	< 0.001
TBSA 3rd degree [%, median (IQR)]	12.0 (4.8–24.8)	3.0 (0.0–9.0)	< 0.001
Inhalation injury [*n* (%)]	25 (29.4)	31 (10.8)	< 0.001
Pre-admission comorbidities^a^			
Hypertension [*n* (%)]	42 (50.6)	140 (52.0)	0.900
Diabetes mellitus [*n* (%)]	20 (24.1)	70 (26.0)	0.775
Respiratory disease [*n* (%)]	22 (26.5)	50 (18.6)	0.122
Current smoker [*n* (%)]	21 (25.3)	51 (19.0)	0.216
Alcoholism [*n* (%)]	18 (21.7)	32 (11.9)	0.031
Illicit drug use [*n* (%)]	5 (6.0)	8 (3.0)	0.196
Major psychiatric illness [*n* (%)]	16 (19.3)	39 (14.5)	0.302
Outcomes			
Operation count [median (IQR)]^a^	3.0 (2.0–5.0)	1.0 (1.0–2.0)	< 0.001
Operation and procedure count [median (IQR)]^a^	8.0 (4.0–16.0)	4.0 (2.0–6.0)	< 0.001
Ventilation [*n* (%)]^a^	71 (85.5)	83 (30.9)	< 0.001
30-day mortality [*n* (%)]	22 (25.9)	21 (7.3)	< 0.001
LOS [d, median (IQR)]^b^	40.0 (29.3–63.8)	18.0 (14.0–25.0)	< 0.001

^a^Data presented for 352 patients (83 sepsis and 269 controls)

^b^Analysis restricted to patients alive until discharge. Numbers may not add to 100 due to rounding

*IQR* interquartile range, *LOS* length of stay, *TBSA* total body surface area

The first episode of sepsis was clinically diagnosed at a median (IQR) of 11 (6–17) d post-burn (Fig. 2c). *Enterococcus* (20.0%), *Pseudomonas* (12.9%), *Klebsiella* (10.6%), and *Staphylococcus* (10.6%) were most often identified at the time of sepsis diagnosis. Approximately 11.8% of patients had multiple positive cultures identified at the time of sepsis diagnosis, and 1.2% were diagnosed with fungal sepsis (Fig. 2d). Investigations into infectious pathogens revealed that Gram-negative sepsis was most common among older adults (50.8%); however, injury characteristics (Additional file 1: Table S4) or the probability of survival (Additional file 1: Fig. S3) did not differ between different pathogen groups.

### Predictors of sepsis diagnosis in older adult burn patients

TBSA, third-degree TBSA, inhalation injury, and alcoholism were significantly associated with sepsis diagnosis in older adult burn patients in univariate models (Additional file 1: Table S5). Each 1% increase in TBSA resulted in an 11% increase in the odds of sepsis diagnosis (*OR* = 1.11, 95% CI 1.08–1.14, *P* < 0.001). Likewise, each 1% increase in third-degree TBSA increased the odds of sepsis diagnosis by 8% (*OR* = 1.08, 95% CI 1.06–1.11, *P* < 0.001), and the presence of inhalation injury increased the odds by approximately 3-fold (*OR* = 3.43, 95% CI 1.89–6.23, *P* < 0.001). Alcoholism also doubled the odds of sepsis diagnosis (*OR* = 2.05, 95% CI 1.08–3.89, *P* = 0.028). Age, female sex, hypertension, diabetes, respiratory disease, smoking status, illicit drug use, and major psychiatric illness were not associated with sepsis diagnosis in older adult burn patients.

In the multivariate model, TBSA and inhalation injury were associated with sepsis diagnosis, whereas age, female sex, and third-degree TBSA showed no association (Fig. 2e). Each 1% increase in TBSA was associated with an 11% increase in the odds of sepsis diagnosis (*OR* = 1.11, 95% CI 1.06–1.15, *P* < 0.001). The presence of inhalation injury doubled the odds of sepsis diagnosis (*OR* = 2.29, 95% CI 1.14–4.60, *P* = 0.020). The model demonstrated good performance, distinguishing between patients with and without sepsis (Omnibus *χ*^2^ = 82.193, *P* < 0.001), explaining 31% of the variance in mortality (Nagelkerke *R*^2^ = 0.305), and showing good fit (Hosmer-Lemeshow *χ*^2^ = 5.897, *P* = 0.659).

### Predictors of mortality in older adult burn patients diagnosed with sepsis

Univariate logistic regression analyses were conducted to examine the associations between various independent variables and mortality among older adult patients diagnosed with sepsis (Additional file 1: Table S6). Each additional day to the first sepsis episode decreased the odds of mortality by 13% (*OR* = 0.87, 95% CI 0.78–0.97, *P* = 0.014). No other variables were associated with mortality among older adult burn patients diagnosed with sepsis.

In the multivariate model, days to first sepsis episode were identified as the only variable associated with mortality (Fig. 2f). Indeed, for each additional day in sepsis onset, the odds of mortality decreased by approximately 16% (*OR* = 0.84, 95% CI 0.73–0.95, *P* = 0.006). Overall, the model demonstrated good performance, distinguishing between survivors and non-survivors (Omnibus *χ*^2^ = 15.617, *P* = 0.016), explaining 30% of the variance in mortality (Nagelkerke *R*^2^ = 0.301), and showing good fit (Hosmer-Lemeshow *χ*^2^ = 7.148, *P* = 0.521).

## Discussion

Burn injuries are one of the leading causes of death in military combat zones, accounting for up to 20% of fatalities [2]. Sepsis is a common and devastating complication in this patient population and a driver of this high mortality [24–26]. However, large-scale epidemiological studies on burn-related sepsis are lacking, which are essential for enhancing our understanding of the distribution, risk factors, and implications of this detrimental complication. To address this gap, we conducted a comprehensive investigation of the incidence and outcomes of sepsis in adult and older adult burn patient populations.

In our study, approximately 1 in 5 burn patients developed sepsis, with similar incidences observed in both adult and older adult burn patients. These incidence rates are consistent with previous studies of adult burn populations with median TBSA similar to those in our cohort, which reported sepsis rates between 14 and 26% [27–29]. Likewise, consistent with the previous literature, we observed a higher incidence of inhalation injury and higher TBSA in adult and older adult sepsis patients compared to controls [27, 29, 30]. Indeed, inhalation injury and burn size increase susceptibility to sepsis by severely compromising the function of the skin’s protective barrier and exacerbating immune dysregulation, significantly impairing the body’s defense mechanisms against invading pathogens [31, 32]. We also determined that TBSA and inhalation injury influenced the risk of sepsis diagnosis, confirming their roles as predictive factors of sepsis for both age groups, even after adjusting for other prognostic covariates within the model. Surprisingly, however, full-thickness burns did not show a significant association with sepsis outcomes, despite the severe tissue damage typically linked to such injuries [33]. We hypothesize that burn size, as indicated by TBSA, may have a more substantial impact on sepsis risk, as larger burns provide a greater surface area for bacterial infiltration. Furthermore, larger burns often lead to more pronounced systemic responses, including immune dysregulation and greater inflammatory responses, which may further elevate sepsis risk [33].

Moreover, burn patients who developed sepsis had a greater prevalence of pre-admission comorbidities associated with greater incidences of systemic infections and immune dysfunction, including alcoholism and illicit drug use among adult sepsis patients and alcoholism in older adult sepsis patients [34, 35]. Intriguingly, alcoholism has been previously reported to be associated with sepsis and septic shock, suggesting that pre-admission comorbidities, such as alcoholism and illicit drug use, could serve as predictive factors for sepsis diagnosis in burn patients, potentially aiding in early identification and intervention [34].

As expected, sepsis diagnosis was linked to a higher mortality, with septic patients experiencing a cumulative incidence of death nearly 4-times higher than non-septic patients in both age groups. Importantly, among patients who were already septic, age, TBSA, third-degree TBSA, and inhalation injury did not further increase the risk of mortality. However, the timing of the onset of the first episode of sepsis was one of the main predictors of mortality. Earlier sepsis onset during hospitalization was found to be more detrimental, while a later onset was associated with better survival. In fact, for each day sepsis was delayed, the odds of mortality decreased by 15% in adults and 16% in older adults. This pattern may be due to a combination of burn-induced metabolic alterations and immune system activation. If sepsis occurs earlier during hospitalization, the patient is likely in a state of immune dysregulation, hemodynamic instability, and metabolic stress, which compromises the body’s ability to combat infections effectively. This period may leave patients more vulnerable to the severe impacts of sepsis, leading to worse outcomes [36]. Indeed, early-onset sepsis may contribute to the development of organ dysfunction within the immediate days post-burn, which, in turn, places patients at higher risk of mortality, consistent with evidence linking early organ dysfunction to poorer outcomes in burn patients [37, 38]. Conversely, if sepsis occurs later during hospitalization, patients may have passed this critical phase, resulting in stabilized organ function, improved perfusion, and robust clinical support to combat infection. Additionally, exposure to bacteria due to the burn injury may prime the innate immune system, leading to more effective antigen presentation and stronger responses to subsequent infections [36, 39, 40]. The later sepsis occurs within burn patients, the more time the immune system has had beforehand to be primed by invading bacteria, thus becoming more effective in combating a large-scale infectious response such as sepsis. Preclinical studies have demonstrated that exposure to bacterial endotoxins, such as lipopolysaccharide, can enhance immune system preparedness and improve inflammatory and cellular responses during sepsis episodes [41, 42]. Therefore, immune priming can lead to more controlled and efficient inflammatory responses during sepsis, reducing the risk of organ failure and mitigating the severity of sepsis. Overall, the timing of sepsis onset plays a pivotal role in determining survival outcomes, with later sepsis onset occurring during a more favorable physiological state and a more prepared immune system, improving survival outcomes.

Interestingly, our study identified female sex as a significant predictor of mortality among adult burn patients diagnosed with sepsis, a difference that diminished among older adults. This is surprising, considering that previous studies in critical care and polytrauma patients have demonstrated that female patients have better prognoses compared to males and different temporal responses in biomarkers [43–45]. However, consistent with our results, female sex has previously been identified as a prognostic factor associated with increased mortality in adult burn patients, though this association tends to decrease with age [46–49]. Although the underlying pathophysiological factors contributing to sex-based disparities in burn patients diagnosed with sepsis have yet to be deciphered, several sex-based physiological differences in immune responses, cytokine secretion, and hormonal fluctuations interacting with burn-induced hypermetabolism may underlie these outcomes [49–53]. Indeed, previous studies have attributed the survival advantage of females in sepsis to increased levels of interleukin-10 (IL-10), an anti-inflammatory mediator [44, 54]. Specifically, estrogen has been implicated in mediating this benefit by suppressing pro-inflammatory cytokines such as interleukin-6 (IL-6), interferon-γ (IFN-γ), and tumor necrosis factor-α (TNF-α), while promoting the release of anti-inflammatory mediators [55–57]. While this attenuation of the hyperinflammatory response may confer advantages in sepsis, it could be detrimental in the context of burn sepsis. These cytokines are crucial for driving the post-burn hypermetabolic response, and estrogen may delay the activation of the pro-inflammatory cascade and the catabolic processes necessary for healing, leading to poorer outcomes in female burn patients. Although prolonged inflammation and hypermetabolism are harmful long-term, they are essential in the acute post-burn phase to mobilize energy reserves for wound healing and to compensate for post-burn sequelae [58]. Specifically, elevated levels of IL-6, IL-1β, and TNF-α during recovery play a crucial role in promoting proteolysis to meet the heightened energy demands of recovery [43–45]. Therefore, our novel finding that adult female burn patients diagnosed with sepsis experience a worse prognosis highlights a need for sex-based considerations in future studies investigating sepsis in burn patients.

Beyond survival, sepsis significantly affected the recovery of adult and older adult burn patients and contributed to the development of post-burn complications. In line with previous studies, septic patients in both age groups had longer hospital stays and were more frequently dependent on mechanical ventilation [8, 59, 60]. Further, they underwent a greater number of operations and procedures, which is also reflected in the higher incidence of skin graft loss in both adult and older adult sepsis patients, as well as a greater incidence of wound infections in adult sepsis patients. Among older adult burn patients, however, burn wound infections affected a similarly high proportion of sepsis and control patients, another testimony of older adults’ impaired immune response [61].

Despite the similar incidence of wound infections in older adults, mortality was markedly higher among sepsis patients. This disparity was likely attributed to the increased injury severity and morbidity of these patients, which was further aggravated by an increased incidence of AKI and pneumonia in older adult sepsis patients. These are common clinical manifestations of sepsis, as infection and multiple organ dysfunction are its defining characteristics [8, 10, 36, 62]. Importantly, in other critical illnesses, short delays in the initialization of antibiotic treatment significantly increased the odds of mortality in sepsis patients [63–65]. Therefore, these findings emphasize the importance and need for future research into early biomarkers of sepsis since early sepsis onset during hospitalization is associated with worse outcomes, and timely adaptation of patient management is crucial for patient survival.

These findings carry important implications for the clinical practice in the care of burn patients in both trauma systems and combat casualty care. First, the identification of specific pre-existing conditions, such as alcoholism and illicit drug use, and injury characteristics as risk factors for the development of sepsis could be integrated into early risk stratification tools upon admission. These patients could be classified as high-risk patients, and a more refined monitoring with prompt therapy escalation upon early signs of sepsis development could help to improve outcomes. Second, the association of earlier sepsis development with increased complication rates suggests a more vulnerable period within the first 10 d post-burn when monitoring efforts should be intensified with adequate fluid resuscitation, infection control, and antimicrobial therapy. This could be enhanced by further biomarker screenings to identify potential candidates that can be implemented in subsequent real-time biomarker monitoring and the integration of predictive algorithms. Third, the observed sex difference in the trajectory of burn sepsis suggests a need for further exploration of the pathophysiology among female burn patients to develop sex-specific management strategies.

Nevertheless, this study has several limitations. First, it is limited by its retrospective design, and while we have taken care to ensure data accuracy and completeness, causal inferences cannot be definitively established. Second, the sources of infection were not considered, which would aid in providing more specific recommendations for burn patient treatment. Third, the lack of data on whether patients received antibiotic treatment prevented us from analyzing the impact of antibiotics on sepsis outcomes. Given the clinical significance of this factor, future studies should investigate the role of antibiotic administration in sepsis management and its potential influence on outcomes in burn patients. Additionally, frailty, an important factor in assessing patient vulnerability and outcomes, particularly for older adults, was not available in our dataset and therefore could not be included in the analysis. Despite these limitations, the strength of our study lies in the large sample size and the reliability of our long-term data, which enhances the generalizability and relevance of our findings. Moreover, it provides an in-depth description of the clinical presentation of sepsis in burn patients and a benchmark for future research into the underlying pathophysiologic mechanisms contributing to the high mortality in this vulnerable population.

## Conclusions

This study provides novel insights into epidemiological factors underlying sepsis in adult and older adult burn patients, with several implications for military medicine, offering insights that can inform better prevention and treatment strategies for burn-related infections in trauma systems and combat casualty care. Increasing age and TBSA, the presence of inhalation injury, and specific comorbidities increase the odds of sepsis diagnosis and should be incorporated for early screening. Additionally, early sepsis onset was established as an independent predictor of mortality in both adult and older adult burn patients, emphasizing the need for increased sepsis monitoring efforts within the first 10 d post-burn. Finally, female sex was independently associated with mortality in adult sepsis patients, indicating the potential need for sex-specific management strategies. Ultimately, future investigations into early sepsis markers, accounting for sex and age differences, are necessary to enhance diagnostic accuracy and facilitate timely therapeutic initiation, thereby improving outcomes for burn patients.

## Supplementary Information

**Additional file 1.** **Fig. S1** Study flow diagram showing patient inclusion and exclusion criteria. **Fig. S2** Differences in survival outcomes among adult burn patients with sepsis, stratified by Gram stain classification of the pathogen identified at diagnosis. **Fig. S3** Differences in survival outcomes among older adult burn patients with sepsis, stratified by Gram stain classification of the pathogen identified at diagnosis. **Table S1** Demographics and injury characteristics of adult sepsis patients based on infectious pathogen classification. **Table S2** Univariate logistic regression analyses in adult burn patients examining the association between various independent variables and sepsis diagnosis. **Table S3** Univariate logistic regression analyses examining the association between various independent variables and mortality in adult burn patients diagnosed with sepsis. **Table S4** Demographics and injury characteristics of older adult sepsis patients based on infectious pathogen classification. **Table S5** Univariate logistic regression analyses examining the association between various independent variables and sepsis diagnosis in older adult burn patients. **Table S6** Univariate logistic regression analyses examining the association between various independent variables and mortality in older adult burn patients diagnosed with sepsis

## Data Availability

Individual participant data that underlie the results reported in this article, after de-identification (text, tables, figures, and appendices), may be shared with investigators whose proposed use of the data has been approved by an independent ethics review committee identified for this purpose. Proposals should be directed to jeschke@hhsc.ca; to gain access, data requestors will need to sign a data transfer agreement.

## Abbreviations

ABA: American Burn Association
AKI: Acute kidney injury
ARDS: Acute respiratory distress syndrome
CI: Confidence interval
HR: Hazard ratio
IQR: Interquartile range
OR: Odds ratio
TBSA: Total body surface area
