# Supplementary material for: Sepsis in burn care: incidence and outcomes
Source: Mil Med Res. 2025 Sep 1;12:55. doi: 10.1186/s40779-025-00643-x (PMC12403279; doi:10.1186/s40779-025-00643-x)
Supplement: Supplementary file 1 — Additional file 1. Fig. S1 Study flow diagram showing patient inclusion and exclusion criteria. Fig. S2 Differences in survival outcomes among adult burn patients with sepsis, stratified by Gram stain classification of the pathogen identified at diagnosis. Fig. S3 Differences in survival outcomes among older adult burn patients with sepsis, stratified by Gram stain classification of the pathogen identified at diagnosis. Table S1 Demographics and injury characteristics of adult sepsis patients based on infectious pathogen classification. Table S2 Univariate logistic regression analyses in adult burn patients examining the association between various independent variables and sepsis diagnosis. Table S3 Univariate logistic regression analyses examining the association between various independent variables and mortality in adult burn patients diagnosed with sepsis. Table S4 Demographics and injury characteristics of older adult sepsis patients based on infectious pathogen classification. Table S5 Univariate logistic regression analyses examining the association between various independent variables and sepsis diagnosis in older adult burn patients. Table S6 Univariate logistic regression analyses examining the association between various independent variables and mortality in older adult burn patients diagnosed with sepsis [file 40779_2025_643_MOESM1_ESM.pdf]

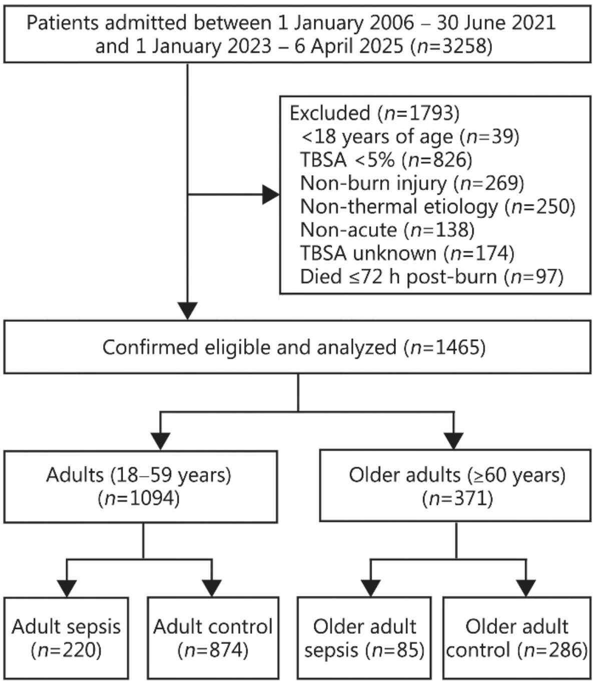

Fig. S1 Study flow diagram showing patient inclusion and exclusion criteria. TBSA total body surface area

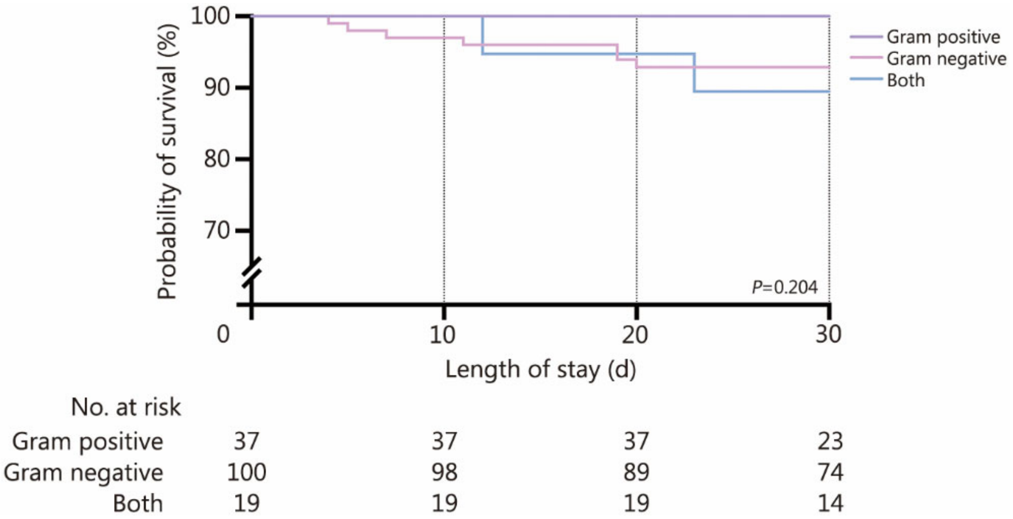

Fig. S2 Differences in survival outcomes among adult burn patients with sepsis, stratified by Gram stain classification of the pathogen identified at diagnosis

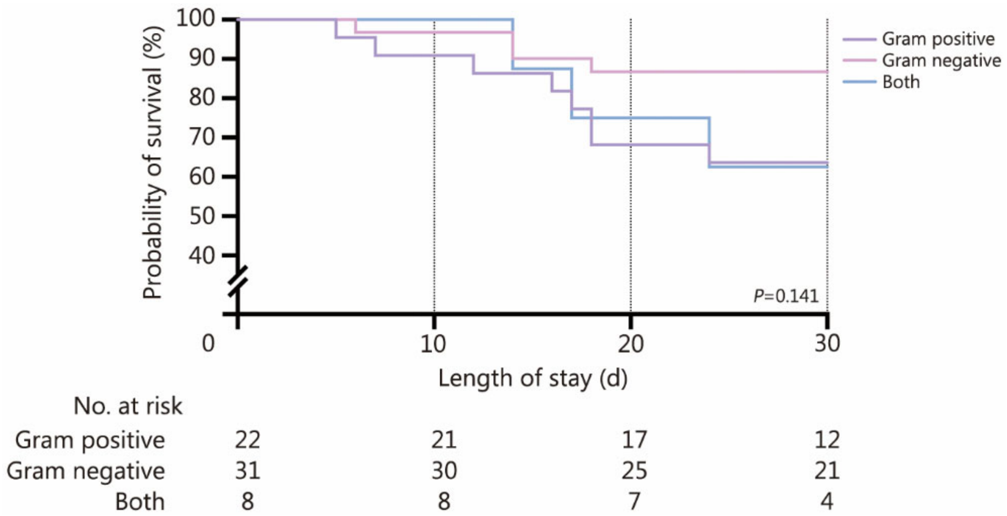

Fig. S3 Differences in survival outcomes among older adult burn patients with sepsis, stratified by Gram stain classification of the pathogen identified at diagnosis

Table S1 Demographics and injury characteristics of adult sepsis patients based on infectious pathogen classification

**Pathogen**	**Gram positive (*n* = 37)**[#]	**Gram negative (*n* = 100)**[#]	**Both (*n* = 19)**[#]	***P*-value**
Age [years, median (IQR)]	46.0 (31.5 – 52.5)	43.0 (36.3 – 52.0)	46.0 (33.0 – 52.0)	0.809
Sex [*n* (%)]				0.430
Male	30 (81.1)	70 (70.0)	14 (73.7)	
Female	7 (18.9)	30 (30.0)	5 (26.3)	
Burn etiology [*n* (%)]				0.989
Scald	3 (8.1)	7 (7.0)	2 (10.5)	
Flame	32 (86.5)	87 (87.0)	16 (84.2)	
Other	2 (5.4)	6 (6.0)	1 (5.3)	
TBSA [%, median (IQR)]	30.0 (20.0 – 45.8)	34.3 (25.3 – 47.4)*	48.5 (35.5 – 57.0)*	0.013
30-day mortality [*n* (%)]	0 (0)	7 (7.0)	2 (10.5)	0.189

[#]Pathogen data was only available for 156 adult sepsis patients. *Denotes adjusted $P < 0.05$. Numbers may not add to 100 due to rounding. *TBSA* total body surface area, *IQR* interquartile range

Table S2 Univariate logistic regression analyses in adult burn patients examining the association between various independent variables and sepsis diagnosis

**Independent variable**	**Unadjusted *OR* (95% CI)**	***P*-value (*OR*)**	**Omnibus tests of model coefficients**		**Nagelkerke R^2**	***P*-value (Hosmer-Lemeshow test)**
			χ^2	***P*-value**		
Age	1.03 (1.01 – 1.04)	< 0.001	14.212	< 0.001	0.020	0.460
Female sex	0.95 (0.68 – 1.33)	0.769	0.086	0.769	0.000	-
TBSA	1.14 (1.12 – 1.15)	< 0.001	407.062	< 0.001	0.490	0.014
TBSA 3rd degree	1.09 (1.08 – 1.11)	< 0.001	239.040	< 0.001	0.313	0.002
Inhalation injury	7.97 (5.72 – 11.11)	< 0.001	152.835	< 0.001	0.206	-
Hypertension	1.42 (0.92 – 2.21)	0.115	2.378	0.123	0.004	-
Diabetes	1.29 (0.72 – 2.31)	0.401	0.679	0.410	0.001	-
Respiratory disease	1.19 (0.64 – 2.21)	0.581	0.297	0.586	0.000	-
Current smoker	0.89 (0.64 – 1.24)	0.498	0.463	0.496	0.001	-
Alcoholism	1.76 (1.23 – 2.52)	0.002	9.088	0.003	0.013	-
Illicit drug use	1.97 (1.38 – 2.81)	< 0.001	13.110	< 0.001	0.019	-
Major psychiatric illness	2.30 (1.61 – 3.27)	< 0.001	19.851	< 0.001	0.029	-

"-" indicates no data. *CI* confidence interval, *TBSA* total body surface area, *OR* odds ratio

Table S3 Univariate logistic regression analyses examining the association between various independent variables and mortality in adult burn patients diagnosed with sepsis

**Independent variable**	**Unadjusted *OR* (95% CI)**	***P*-value (*OR*)**	**Omnibus tests of model coefficients**		**Nagelkerke R^2**	***P*-value (Hosmer-Lemeshow test)**
			χ^2	***P*-value**		
Days to first episode of sepsis	0.83 (0.72 – 0.96)	0.013	9.364	0.002	0.125	0.752
Age (years)	1.06 (1.00 – 1.12)	0.052	4.390	0.036	0.049	0.408
Female sex	4.18 (1.48 – 11.81)	0.007	7.193	0.007	0.079	-
TBSA	1.03 (1.00 – 1.06)	0.075	3.131	0.077	0.035	0.168
TBSA 3rd degree	1.03 (1.01 – 1.06)	0.008	7.043	0.008	0.078	0.719
Inhalation injury	1.14 (0.41 – 3.19)	0.799	0.065	0.798	0.001	-
Hypertension	1.42 (0.38 – 5.29)	0.604	0.254	0.615	0.003	-
Diabetes	-	-	2.563	0.109	0.029	-
Respiratory disease	2.24 (0.46 – 11.00)	0.321	0.843	0.359	0.009	-
Current smoker	1.23 (0.41 – 3.70)	0.714	0.131	0.717	0.001	-
Alcoholism	2.53 (0.90 – 7.17)	0.080	2.900	0.089	0.033	-
Illicit drug use	0.62 (0.17 – 2.28)	0.475	0.555	0.456	0.006	-
Major psychiatric illness	0.58 (0.16 – 2.11)	0.407	0.756	0.385	0.009	-

"-" indicates no data. *CI* confidence interval, *TBSA* total body surface area, *OR* odds ratio

Table S4 Demographics and injury characteristics of older adult sepsis patients based on infectious pathogen classification

**Pathogen**	**Gram positive (*n* = 22)#**	**Gram negative (*n* = 31)#**	**Both (*n* = 8)#**	***P*-value**
Age [years, median (IQR)]	69.0 (63.0 – 81.0)	72.0 (64.0 – 80.0)	79.0 (68.5 – 83.3)	0.423
Sex [*n* (%)]				0.121
Male	17 (77.3)	21 (67.7)	3 (37.5)	
Female	5 (22.7)	10 (32.3)	5 (62.5)	
Burn etiology [*n* (%)]				0.925
Scald	4 (18.2)	4 (12.9)	1 (12.5)	
Flame	17 (77.3)	25 (80.7)	7 (87.5)	
Other	1 (4.6)	2 (6.5)	0 (0)	
TBSA [%, median (IQR)]	22.0 (15.0 – 30.3)	26.0 (17.0 – 35.0)	30.0 (8.0 – 43.3)	0.758
Mortality [*n* (%)]	8 (36.4)	4 (12.9)	3 (37.5)	0.098

#Pathogen data was only available for 61 older adult sepsis patients. Numbers may not add to 100 due to rounding. *TBSA* total body surface area, *IQR* interquartile range

Table S5 Univariate logistic regression analyses examining the association between various independent variables and sepsis diagnosis in older adult burn patients

**Independent variable**	**Unadjusted *OR* (95% CI)**	***P*-value (*OR*)**	**Omnibus tests of model coefficients**		**Nagelkerke R^2**	***P*-value (Hosmer-Lemeshow test)**
			χ^2	***P*-value**		
Age	1.01 (0.98 – 1.03)	0.698	0.150	0.699	0.001	0.712
Female sex	0.83 (0.49 – 1.40)	0.474	0.521	0.470	0.002	-
TBSA	1.11 (1.08 – 1.14)	< 0.001	74.332	< 0.001	0.275	0.214
TBSA 3rd degree	1.08 (1.06 – 1.11)	< 0.001	50.517	< 0.001	0.196	0.843
Inhalation injury	3.43 (1.89 – 6.23)	< 0.001	15.601	< 0.001	0.062	-
Hypertension	0.94 (0.58 – 1.54)	0.818	0.053	0.818	0.000	-
Diabetes	0.90 (0.51 – 1.60)	0.725	0.125	0.724	0.001	-
Respiratory disease	1.58 (0.89 – 2.81)	0.120	2.340	0.126	0.010	-
Current smoker	1.45 (0.81 – 2.59)	0.212	1.513	0.219	0.006	-
Alcoholism	2.05 (1.08 – 3.89)	0.028	4.605	0.032	0.020	-
Illicit drug use	2.09 (0.67 – 6.58)	0.207	1.489	0.222	0.006	-
Major psychiatric illness	1.41 (0.74 – 2.68)	0.296	1.056	0.304	0.005	-

"-" indicates no data. *CI* confidence interval, *TBSA* total body surface area, *OR* odds ratio

Table S6 Univariate logistic regression analyses examining the association between various independent variables and mortality in older adult burn patients diagnosed with sepsis

**Independent variable**	**Unadjusted *OR* (95% CI)**	***P*-value (*OR*)**	**Omnibus tests of model coefficients**		**Nagelkerke R^2**	***P*-value (Hosmer-Lemeshow test)**
			χ^2	***P*-value**		
Days to first episode of sepsis	0.87 (0.78 – 0.97)	0.014	9.194	0.002	0.185	0.513
Age	1.00 (0.94 – 1.05)	0.888	0.020	0.888	0.000	0.725
Female sex	1.17 (0.41 – 3.34)	0.774	0.082	0.775	0.001	-
TBSA	0.99 (0.95 – 1.02)	0.479	0.523	0.469	0.009	0.395
TBSA 3rd degree	1.00 (0.96 – 1.03)	0.768	0.089	0.766	0.002	0.727
Inhalation injury	0.63 (0.20 – 1.96)	0.426	0.661	0.416	0.011	-
Hypertension	1.85 (0.67 – 5.09)	0.234	1.448	0.229	0.026	-
Diabetes	2.56 (0.87 – 7.57)	0.088	2.828	0.093	0.049	-
Respiratory disease	1.15 (0.38 – 3.47)	0.804	0.061	0.805	0.001	-
Current smoker	0.90 (0.28 – 2.85)	0.856	0.033	0.855	0.001	-
Alcoholism	0.81 (0.23 – 2.79)	0.735	0.118	0.732	0.002	-
Illicit drug use	-	-	3.024	0.082	0.053	-
Major psychiatric illness	1.45 (0.44 – 4.80)	0.544	0.358	0.549	0.006	-

"-" indicates no data. *CI* confidence interval, *TBSA* total body surface area, *OR* odds ratio
